# Disease-related disgust promotes antibody release in human saliva

**DOI:** 10.1016/j.bbih.2022.100489

**Published:** 2022-07-14

**Authors:** Judith K. Keller, Clemens Wülfing, Jannes Wahl, Esther K. Diekhof

**Affiliations:** a Neuroendocrinology and Human Biology Unit, Department of Biology, Faculty of Mathematics, Informatics and Natural Sciences, Institute for Animal Cell- and Systems Biology, Universität Hamburg, Hamburg, Germany; b Interdisciplinary Neurobiology and Immunology, Department of Biology, Faculty of Mathematics, Informatics and Natural Sciences, Institute for Animal Cell- and Systems Biology, Universität Hamburg, Hamburg, Germany

**Keywords:** Behavioral immune system, Secretory IgA, Disgust, Prime, Disease, Avoidance

## Abstract

The behavioral immune system (BIS) comprises manifold mechanisms, that may assist the physiological immune system (PIS) in counteracting infection and can even reduce the risk of contagion. Previous studies have found initial evidence for possible interactions between the two systems. However, most of these findings were correlative and have not been replicated. Further, none of these studies examined whether disease stimuli that indicate an enhanced airborne transmission risk may trigger a different immune response in comparison to stimuli that predominantly evoke core disgust. In the present study, we employed a video-priming approach to get further insight in the influence of the perception of disgust- and disease-related stimuli on the rapid physiological immune response, as indicated by changes of secretory immunoglobulin A (S-IgA) in saliva. We created three video primers that represented different categories of disgust- and/or disease-associated content. Two of the videos showed disease-related situations that were associated with contagious respiratory virus infections, varying in concealment of aerosols. The third video incurred no heightened airborne contagion risk, but comprised situations that are known to elicit core disgust, such as rotten foods, decaying animal carcasses, or cockroaches. A fourth video acted as control showing landscape impressions. The different video primers varied in their contagion risk and disgust-evoking potential. Given the role of S-IgA in the mucosal immune defense, we expected differences in the S-IgA response between the two videos indicating a heightened airborne contagion risk and the core disgust video, with the highest S-IgA to occur after the aerosol video. For this, we used the data of 107 healthy participants in a between-subjects design with the four video primers. We found a significant increase of S-IgA in response to both the disease- and the disgust-related videos, which correlated positively with the perceived contagion risk of the displayed situations. Nevertheless, there was no significant difference in the increase between the three disease- and disgust-related videos. We also found that people with a high contamination disgust produced less S-IgA in such situations, which is a hint for a compensating relationship between the BIS and PIS. Our observations suggest that the mere visual perception of videos showing realistic situations of an increased contagion risk can elicit a heightened release of salivary antibodies.

## Highlights

• Realistic videos of respiratory diseases are efficient to trigger immune responses.
• Secretory Immunoglobulin A (S-IgA) in saliva increases after video-priming.
• S-IgA increase is positively correlated with perceived contagion risk.
• S-IgA increase is inversely correlated with the trait contamination disgust.

## 1 Introduction

The physiological immune system (PIS) has evolved due to the constant pathogen threat in the environment. While both the unspecific, innate and the specific, adaptive immune system are highly effective, most functions of the PIS are very resource consuming and can have negative consequences when misdirected (McDade, 2003). This led scientists to propose the theory of a behavioral immune system (BIS), first described as such by (Schaller and Duncan, 2007). The BIS comprises mechanisms that aim to proactively avoid pathogens even before such pathogens are coming in contact with the organism. Thus, activation of the BIS might reduce the necessity to activate the PIS.

As a complex system, the BIS may detect potential pathogens, and trigger defensive responses like disgust, avoidance behavior, social exclusion and sickness behavior (Schaller and Park, 2011). The ongoing Covid-19 pandemic has shown that particularly social avoidance can be very effective in reducing contagion risk. Yet, the various mechanisms of the BIS may also incur costs for the individual. While social distancing has proven as an effective method to reduce the spread of Sars-CoV-2 (Qian and Jiang, 2020), it has led to economical (Tuzovic and Kabadayi, 2021), social and psychological distress in many individuals (Marroquín et al., 2020). Even on the small scale, pathogen avoidance carries costs of lost opportunity (e.g., the reduction of social contacts can lead to lower mating chances), hence researchers have suggested that the BIS and PIS are interconnected to optimize the cost/benefit ratio of both (Gangestad and Grebe, 2014; Oaten et al., 2009; Schaller and Park, 2011). As the BIS is mainly triggered by sensory cues (e.g.: visual stimuli and auditory), the interaction between the two systems would most likely be part of the neuro-immune-axis, acting either over endocrine mechanisms or the autonomic nervous system (for review Wrona, 2006). The exact route by which the brain and the immune system interact in this context has not been investigated. Nevertheless, to do so it is important to find a method to reliably trigger the BIS-PIS interaction, before moving experiments into neuro-pathway fields (e.g., functional neuroimaging).

Previous studies found initial evidence for possible interactions between the BIS and the PIS: (1.) avoidance behavior increased after recent activation of the PIS (Miller and Maner, 2011), (2.) behavioral immune responses were lower in men with a proposedly more effective physiological immune response (Kandrik et al., 2017), and (3.) higher germ aversion predicted lower chronic basal inflammation (Gassen et al., 2018). However, most of these findings were correlative and findings have not been replicated (Tybur et al., 2020).

A more direct measure to investigate the relationship between the PIS and BIS is to experimentally confront participants with disgust evoking stimuli, in order to provoke behavioral and physiological immune responses. Disgust may have evolved as a response to objects that represent a potential threat of (infectious) diseases (Curtis et al., 2004; Tybur et al., 2009). The emotion of disgust also correlates with avoidance behavior (Campbell et al., 2020; Dorfan and Woody, 2011). Two studies from the group of Stevenson found an increase in various immune parameters after presentation of disgust evoking stimuli, such as pictures of rotten food, animals and wounds. These included a rise in body temperature, Tumor Necrosis Factor alpha (TNF-a) and albumin levels relative to control stimuli (Stevenson et al., 2011, 2012). In another study, Schaller et al. (2010) assessed the relationship between the BIS and the PIS from a somewhat different methodological angle. They used pictures of people showing disease symptoms. These included morphological (pox, skin lesions) and behavioral (sneezing, coughing) characteristics of various illnesses. While participants were not more disgusted by the disease-related than the control pictures, the study nevertheless found an increase in the interleukin-6 (IL-6) blood concentration after the disease stimuli.

Setting the focus on the mucosal immune response Brown et al. (2014) measured the change of secretory immunoglobulin A (S-IgA) in saliva. While they initially found an increase in S-IgA after showing disease (people infected with diseases, showing symptoms like sores, fever, paleness) and mutilation stimuli (lacerations, burns and amputations), they failed to replicate their own results in a second study, which was published in the same paper. S-IgA has also been measured in studies that presented disgust-evoking stimuli, with no direct disease-association (surgery video, rotten food, etc.). However, contrary to expectation, S-IgA rather seemed to decrease in these studies (Bosch et al., 2001; Stevenson et al., 2011, 2012). In an attempt to disentangle if disgust- and disease-related stimuli rely on the same pathways, Stevenson et al. (2015) therefore compared the immune responses after disease- and disgust-related stimuli. For this purpose, they created two sets of images, of which one was classified as disgusting, but not directly disease-related (e.g., picture of garbage, dead animals, mutilated body parts), while the other was intended to evoke disease-related concerns without being overly disgusting (e.g., pictures of hospital ward, x-rays, sneezing). Both sets of images failed to increase S-IgA and other salivary immune parameters (TNF-α, albumin, cortisol), part of which were previously identified to be disgust-sensitive (Stevenson et al., 2011). Only after a secondary analysis, Stevenson et al. (2015) found an increase in S-IgA and TNF- α in a subgroup of people with higher-than-average self-reported disgust sensitivity.

In the present study, we took a similar approach as Stevenson et al. (2015) to get further insight in the effect of disgust- and disease-related stimuli on the physiological immune response. One may argue that the nature of the immune response probably depends on the class of pathogens presented (Bradshaw and Gassen, 2021). Therefore, we decided to use disease-associated stimuli, which were more realistic and were also more specifically associated with respiratory contagious pathogens than the stimuli used in previous studies. In contrast to our stimuli, the stimuli of previous studies, comprised images of x-rays or hospital hallways (Stevenson et al., 2015) or of sick people, who mostly exhibited no sign of respiratory illnesses (e.g., people with skin rashes or open wounds) (Brown et al., 2014; Schaller et al., 2010). For this purpose, and in contrast to previous studies, our stimulus material also included short videos of people with visible signs of sickness or of disgust-evoking scenes. Further, we created three different stimulus sets that each represented a different level of contagion risk and varied in their disgust-evoking potential. These included two disease-related sets of people displaying obvious symptoms of respiratory diseases (e.g., the flu, a common cold) that either directly or indirectly implied an increased airborne contagion potential, i.e., the aerosol and the concealed contagion category. The aerosol category thereby included videos and pictures of openly sneezing people, most of them oriented towards the camera, with more or less visible aerosol spread like flying droplets or sputum. Conversely, in the concealed contagion category we showed people with flu-like symptoms (e.g., videos and pictures of people looking pale and lying in bed, obviously suffering from headache and fever, or having a red nose). Some of them were also sneezing or coughing, but they were concealing it by covering their nose or mouth. The third stimulus set focused on sickening disgust-evoking stimuli (core disgust category; e.g., videos and pictures of parasites, rotten foods, dead animals) that would need direct body-contact (e.g., by being touched or actively ingested) to induce an illness. Finally, we also included a control video without an association to disgusting or disease-evoking stimuli, which consisted of landscape videos (e.g., city panoramas, aerial views of busy crossroads).

Since the pathogens associated with respiratory diseases enter the body through mucosal tissues of the respiratory tract, we focused on the mucosal immune system of the oral cavity, specifically the change of salivary S-IgA. S-IgA is the main mucosal immunoglobulin and plays an important role in the first-line-immune-defense against pathogens that enter the body through mouth or nose (Woof and Mestecky, 2015). It is constantly secreted into saliva at a base rate, and can be rapidly upregulated by (para-)sympathical (Carpenter et al., 1998) and mechanical stimulation (Proctor and Carpenter, 2001). S-IgA is part of several immunological processes such as immune exclusion, i.e., the binding of antigens and prevention of attachment to epithelia cells, and intracellular neutralization, i.e. the neutralization of viral replication in epithelial cells, which play an important role in infection immunity (Corthésy, 2013; Strugnell and Wijburg, 2010). The responsiveness of the S-IgA secretion to visual stimulation by disease- and disgust-related stimuli has been assessed previously (Bosch et al., 2001; Stevenson et al., 2011, 2012, 2015; Brown et al., 2014), but results have been inconsistent.

In our study, young healthy participants watched one of the four videos and answered an online survey to assess inter-individual state and trait differences in the BIS (see details below). Saliva samples were collected at baseline and twice after the video, from which changes in S-IgA were assessed. Given the well-established role of S-IgA in the mucosal immune defense (Woof and Mestecky, 2015), we expected that the participants in the aerosol video group would experience the spreading aerosol directed towards them as a higher threat of contagion, compared to the concealed contagion disease stimuli and also to the core disgust stimuli. Consequently, we expected the highest S-IgA response in the aerosol group compared to the concealed contagion group and the other two groups (i.e., core disgust and control group). Based on previous studies, investigating the S-IgA response to disgust evoking stimuli (Bosch et al., 2001; Stevenson et al., 2011, 2012), we expected the secretion of S-IgA to go down in the group of participants, who watched the core disgust primer. This would also be consistent with the assumption that the mere visual perception of parasites or rotten food is not expected to trigger a mucosal immune response in the oral cavity (Bradshaw and Gassen, 2021).

To shine more light on inter-individual differences in the behavioral immune system we also utilized established questionnaires that capture trait as well as state disgust (Disgust Scale-Revised (Olatunji et al., 2007),) and self-reported disease vulnerability (pVtD, (Duncan et al., 2009). We expected changes in S-IgA depending on these traits in interaction with the category of the video primer. Based on the finding that S-IgA increases in individuals with higher than average trait disgust (Stevenson et al., 2015), we expected that trait disgust may be associated with a stronger increase in S-IgA, especially after the core disgust primer. Previous studies showed that people with a high self-reported vulnerability to disease show a lower physiological immune response (reduced inflammatory markers in blood, decreased general fitness of the immune system) (Gassen et al., 2018; Kandrik et al., 2017). This supports the theory that the BIS may have evolved to relieve the PIS. Based on this, we expected that participants with a higher pVtD score should exhibit a lower response in S-IgA secretion, and particularly so with regard to the videos with disease-related content.

## 2 Materials & methods

### 2.1 Participants

In our pre-registered https://osf.io/9hxpt/) between-subjects design we confronted the participants with three different disease- and/or disgust-related stimulus sets. In addition to that, we also included a control group, who watched a video with neutral content (landscape impressions and city panoramas). A power analysis with G*power (Faul et al., 2007) indicated that a sample size of 96 participants (24 individuals in each group) would provide sufficient power (1-β = 0.90) to detect a large effect of f = 0.40 with α = 0.05. To compensate for dropouts we tested five additional subjects per condition. We recruited 116 participants (47 m/69 f) on the university campus, through online advertisements and via social media. We only invited healthy individuals to participate, who (a) indicated German as a native language, (b) were of legal age but not older than 35 years, (c) were not smoking regularly, (d) had no hormonal, genetical, or other chronical diseases, (e) had not been vaccinated in the last 3 weeks, and (f) were willing to participate online for the approximate duration of 1 h. Female participants were only included, if they used hormonal contraception. Data collection took place between May and October of 2021. Participants received a financial reward of 12 Euros for completing the appointment. We obtained informed consent from all participants and the procedure was approved by the local ethics committee *“Ethikkommission der Ärztekammer Hamburg”* and conformed with the Declaration of Helsinki.

### 2.2 Stimuli

The participants were randomly assigned to one of four videos (i.e., video primers). These videos differed in their disgust-evoking potential and their disease-related content. The aim was to compare three disgust- and/or disease-associated primers, which triggered different degrees of disgust and fear of contagion. All videos showed a combination of short video clips and pictures that were assembled in a video of 1:20 min length. In order to achieve a sufficient priming effect, each video was repeated once (total video length = 2:40 min). The videos consisted of royalty free material from pages like pexels.com and pixaby.com, while some were bought of istockphoto.com. The stills in the Core Disgust video were taken from the DIRTI-Database (Haberkamp et al., 2017). Most of the openly sneezing people were from the *“Bless-you”* video, by Ulf Lundin. For a detailed description, see Supplementary Tables 1–4.

*Aerosol Disease Video (A):* This video was intended to trigger a high level of disgust and high fear of contagion. It comprised video clips and pictures of people, who were sneezing unconcealed, either directly into the camera or in the vicinity, whereby some visibly emitted aerosols.

*Concealed Contagion Disease Video (CC):* This video showed people sneezing without emitting aerosols (e.g., sneezing into a tissue). Other people in the video showed visible signs of sickness, such as looking feverishly or laying sick in bed. We predicted this video to elicit less disgust and a medium to high fear of contagion, compared to the Aerosol Disease Video.

*Core Disgust Video (CD):* Presuming that non-airborne disease threats trigger a different immune response, we created a video showing rotten food, dead animals with maggots, rats, and other disgusting items. Through this video, we tried to elicit a similar disgust response as in the Aerosol Disease Video, yet with a significantly lowered fear of contagion.

*Control Video (C):* In this video, we combined video clips and photos of buildings, skylines, traffic intersections and other landscape views. This video was intended to trigger no disgust- or disease-related responses.

### 2.3 Online surveys

Our participants underwent online surveys (see 2.5 Procedure below) that were programmed with the software LimeSurvey and Inquisit 6 (Milliseconds, 2021). The Zoom application was further used for interactions between the participant and the experimenter in the breaks between the different tasks. Throughout the experiment, they evaluated their trait disgust, vulnerability to disease and changes of state disgust, mood, etc. related to the video in the following Questionnaires.

#### 2.3.1 Before the video

*Mood Scale:* Participants rated how they feel, answering 24 questions adapted from the German MDBF (Mehrdimensionaler Befindlichkeitsfragebogen) mood-scale from 1 to 5 (1 = not at all; 5 = a lot). For example, tired, satisfied, happy, nervous, etc. (Steyer et al., 1997).

*Disgust Scale-Revised (DS-R):* Participants evaluated their trait disgust sensitivity on the modified version of the Disgust Scale established by Haidt et al. (1994) and revised by Olatunji et al. (2007). This scale consisted of 17 items, eight of these were true-false items with statements like “*I might be willing to try eating monkey meat, under some circumstances.*“, for which participants indicated their agreement on a 5-point Likert-scale (from 0 = ‘*Strongly disagree*’ to 4 = ‘*Strongly agree*’). The rest of the items had the participant rate situations from 0 = ‘*Not disgusting at all*’ to 4 = ‘*Extremely disgusting*’, for example, “*While you are walking through a tunnel under a railroad track, you smell urine.*”. The resulting score were then categorized into the Core-Disgust-Score (12 items), and the Contamination-Disgust-Score (5 items).

*Perceived Vulnerability to Disease (pVtD):* Participants evaluated their perceived Vulnerability to Disease, by using a 15-item self-report instrument designed by Duncan et al. (2009). These 15 items included statements like “*In general, I am very susceptible to colds, flu and other infectious diseases.*” and “*I prefer to wash my hands pretty soon after shaking someone's hand.*”. The participants had to evaluate each item with a 7-point Likert-scale (1 = ‘*Strongly disagree*’ to 7 = ‘*Strongly agree’*). The resulting score was further categorized into the sub-scores Germ-Aversion (8 items) and the Perceived-Infectability- (7 items).

#### 2.3.2 After the video

*Video-Questionnaire:* Participants had to answer questions about the video content. We first asked three questions that required a recall of details, such as “*How many elderly men were portrayed in the video?*”. The participants had a choice between five options, such as “*None*”, or “*Only 1 elderly man*”. Furthermore, participants were shown 15 pictures of which 10 were screenshots from the video previously shown, while 5 were not shown in the video. The participants were asked, “*Was this person/situation portrayed in the video?*” and had to choose between the options “*Yes*” and “*No*”. These questions allowed us to implicitly evaluate whether participants had payed attention to the details of the video, participants that answered less than 50% correct would be excluded.

Lastly participants answered explicit questions on how much attention they had paid during the video and how realistic they would rate them. For this we used nine statements, such as “*I was distracted during the video*.” and “*If this was a real situation I would have become sick.*” (Contagion risk question), which they answered on a 5-point Likert scale form −2 ’*Completely incorrect*’ to +2 ’*Completely correct*’. For analysis this scale was converted to a span from 0 to 4.

*Modified Differential Emotions Scale (mDES):* Participants were further asked to recall the explicit feelings they experienced while watching the video. For this “absolute recall task”, we asked them how they felt, while watching the video using 6 statements, such as “*How strong was your feeling of disgust, antipathy and revulsion while watching the video?*”. The statements about feelings had to be rated on a 9-point Likert-scale from 0 “not at all” to 8 “completely”(Brandenburg and Backhaus, 2015; Fredrickson, 2013).

*Relative-Feelings:* Participants were asked for the relative change in their feelings compared to their emotional state before the video, using another 6 statements, such as “*After the* *video I* *feel weaker and sickly.*”, which they answered on a 5-point Likert-scale from −2 *’I feel much less like that than before the video*‘ to +2 *’I feel a lot more like that than before the video*‘.

Both the absolute (mDES) and the relative recall of feelings depicted an identical number of negative (e.g., stress, disgust) and positive feelings (e.g., amusement, inspiration) and reflected emotional state after the video.

*Evaluation of Stimuli:* Participants evaluated the amount of disgust in relation to the scenes and picture in the videos. For this, we used 37 screenshots from all four videos independent of whether they had been shown to the respective participant (each participant watched only one of the videos, which was randomly assigned). The participants had to decide on how they felt, when watching the situation or person on a given screenshot. For this, they used a 7-point Likert-scale from 1 “*no disgust at all*” to 7 “*extremely strong disgust*”. Through these questions, we were able to evaluate the actual impact of the stimuli in terms of their disgust potential, and were also able to assess the influence of the video prime on subsequent stimulus evaluation. For results of every single stimuli, see Supplementary Tables 1–4.

### 2.4 Saliva samples

The participants received a kit for saliva collection at home. It was sent by mail, since the restrictions related to the COVID-19-pandemic precluded tests in our computer lab at the university. The kit included three 2 ml Eppendorf Tubes and an instruction of proper saliva sample collection. Once the experiment was completed, the envelope with the samples was immediately sent back to the institute. During the test session, the participants were asked to take their saliva samples at pre-defined time points during the surveys. The experimenter, who was blind to the participant's condition, instructed and monitored the saliva sample collection via Zoom calls. The experimenter also answered any study-related questions prior and after the test session via Zoom. However, the subject was left in private while he/she watched the video prime and filled in the online-survey (questionnaires and demographic data). Upon arrival at the institute, the saliva samples were frozen at −20 °C. For analysis, the frozen saliva samples were sent on dry ice to the MVZ Laboratory Vo*lkmann*, Karlsruhe, Germany. There, an immuno-nephelometric analysis to determine the concentration of S-IgA in saliva was performed on the Atellica® NEPH 630 System S-IgA.

### 2.5 Procedure

The computer test was conducted in the afternoon (between 1pm and 5pm). Previous evidence suggests that S-IgA shows its daily peak in the morning and then drops to a stable level in the afternoon (Shirakawa et al., 2004). In the beginning of the test session, participants were informed about the general purpose of the study, the opportunity to abort data collection at any time, as well as aspects concerning anonymity and safety. Then, participants provided demographic data on aspects such as age, gender, and current state of health. They also reported, whether they had been exposed to any stressors, such as smoking, sports, alcohol within the last 48 h, as well as current and previous diseases, before moving on to the Mood Scale. After that, participants collected the first saliva sample (baseline sample). The experimenter documented the time participants needed to collect 1.5 mL of saliva. The duration of sample collection was also documented for the next two samples. On average, participants needed x̅ = 3.88 min/sample. Afterwards the participants moved on to answer the DS-R and pVtD. After completion, the participant was linked to the online software Inquisit. There, each participant was randomly assigned to one of the four video primers. The second saliva sample was taken immediately after the end of the video primer. It was followed by the Attention, the mDES and the Relative-Feelings questionnaires. After this last survey (x̅_duration_ = 5.5 min), participants were asked to give the third and last saliva sample. Then, participants completed the evaluation of the stimuli (also see Fig. 2).

### 2.6 Data analysis

For data analysis, we calculated S-IgA concentration against the time it took the subjects to fill the tube with 1.5 ml of saliva [(mg/dl)/min]. All data was tested for deviation from a normal distribution using the Kolmogorov-Smirnov test with a statistical threshold of p = .050. Since all tests were significant, we used non-parametric post-hoc tests.

Firstly, we used Kruskal-Wallis-tests, with Primer as independent and post-video evaluation scores as dependent variables to assess how primer category affected the evaluation of the video content. Again, we utilized Mann-Whitney-U tests as post-hocs.

Secondly, we assessed whether the increase in S-IgA was affected by the category of the video primers. For this, we utilized a general linear model for repeated measures (GLM) with Sample (Baseline, Sample 2 and Sample 3) as within-subject factor and Primer (Aerosol, Concealed Contagion, Core Disgust and Control) as between-subjects factor. We used the Mann-Whitney-U test as post-hoc test. All post-hoc analyses included Bonferroni adjusted p-values (p_a_).

For the assessment of correlations between the video-evoked increase in S-IgA and (1.) state disgust, (2.) trait disgust, (3.) trait VtD, and (4.) and fear of contagion, we employed the spearman rank correlation. All statistical analyses were performed in IBM SPSS Statistics 27. Figures were created with R Studio and MS-Excel.

## 3 Results

We excluded data of 9 participants, who were exposed to more than three of the stressors listed in the initial survey in the 48 h before the test. The final analysis was therefore based on data of 107 participants, with an average age of 24.72 years (σ = 3.60 years). Of these, 27 participants received the Concealed Contagion Disease Video (16 f/11 m), another 27 watched the Core Disgust Video (18 f/9 m) or the Control Video (16f/11 m), and the remaining 26 participants received the Aerosol Disease Video (14f/12m).

### 3.1 Evaluation of the video primers

In order to test, whether participants indeed differentially perceived the three videos in terms of their disgustingness and contagion risk we analyzed two survey questions, which were asked after the video. The first question was part of the mDES and asked: “*How strong was your feeling of disgust, antipathy and revulsion while watching the video?*”. The Kruskal-Wallis-Test showed that the four video primers differed significantly in disgustingness (H = 56.63; df = 106; p < .001, η^2^_H_ = 0.492, see Fig. 3a). While the Aerosol Disease Video and the Core Disgust Video elicited an equal amount of disgust (A vs. CD: U = −5.98, p = .477, p_a_ = 1, η = 0.010), the Concealed Contagion Disease Video elicited less disgust than these two (CC vs. A: U = 23.92, p = .004, p_a_ = .025, η = 0.197; CC vs. CD: U = −29.90, p < .001, p_a_ = .002, η = 0.332). Furthermore, all three disgust- or disease-related primers triggered higher disgust ratings than the Control Video (C vs. A: U = 50.09, p < .001, p_a_ < .001, η = 0.626; C vs. CC: U = 26.17, p = .002, p_a_ = .009, η = 0.353; C vs. CD: U = 56.07, p < .001, p_a_ < .001, η = 0.721).

Regarding the contagion risk associated with the stimuli in the video, we asked participants to rate the following statement in the Video-Questionnaire: “*If this was a real situation I would have become sick.*”. The Kruskal-Wallis-Test showed that the four primers differed significantly in the perceived contagion risk (H = 25.39; df = 106; p < .001, η^2^_H_ = 0.217, see Fig. 3b). We found that all three disgust- or disease-related primers triggered a higher fear of contagion compared to the Control video (C vs. A: U = 33.10, p < .001, p_a_ < .001, η = 0.292; C vs. CC: U = 36.80, p < .001, p_a_ < .001, η = 0.439; C vs. CD: U = 25.93, p = .001, p_a_ = .009, η = 0.231). However, there was no significant difference in the rating between the three primers themselves (CD vs. A: U = 7.17, p = .382, p_a_ = 1, η = 0.016; CD vs. CC: U = 10.868, p = .181, p_a_ = 1, η = 0.036; A vs. CC: U = −3.694, p = .650, p_a_ = 1, η < 0.001).

## 4 Primer-induced changes in S-IgA

Using a 4 (Primer) x 2 (Sample) GLM, we found a significant main effect of Sample (F(1, 103) = 15.75, p < .001, η_p_^2^ = 0.133) as well as an interaction of Sample and Primer (F(3,103) = 3.09, p = .030, η_p_^2^ = 0.083) on S-IgA. To further investigate these effects, we split the data by Primer and performed Wilcoxon signed-rank tests between the Baseline sample and Sample 2, which was collected directly after the video. We found that watching any of the three disgust- and/or disease-related primers led to a significant increase of S-IgA after the video, while the control primer did not (see Table 1, Fig. 4).

**Table 1:** Descriptive statistics of the comparison of S-IgA [(mg/dl)/min] between Baseline and Sample 2 for the different primers.

Stimuli	n	x̅_Baseline_	x̅_Sample2_	σ_Basline_	σ_Sample2_	z	p
Aerosol	26	2.26	4.14	2.23	4.75	2.58	.010 *
Concealed Contagion	27	1.28	2.57	1.38	3.18	3.14	.002 **
Core Disgust	27	1.60	2.31	1.72	2.79	2.21	.027 *
Kontrolle	27	1.59	1.50	2.42	1.56	.79	.428 -

To further compare these increases between video primers, we calculated the difference between Sample 2 and the Baseline sample (ΔS-IgA). Yet, we found that none of the four primers showed a significantly larger increase than any of the others after the Bonferroni correction. (A vs. CC: U = 341.0, p = .859, p_a_ = 1, η < 0.001; A vs. CD: U = 317,5,p = .551, p_a_ = 1, η = 0.007; A vs. C: U = 256,0, p = .091, p_a_ = .546, η = 0.054; CC vs. C: U = 239.0, p = .030, p_a_ = .180, η = 0.087; CC vs. CD: U = 313.0, p = .373, p_a_ = 1, η = 0.015; CD vs. C: U = 279.5, p = .141, p_a_ = .846, η = 0.040; also see Supplement Fig. 1).

**Fig. 1:**
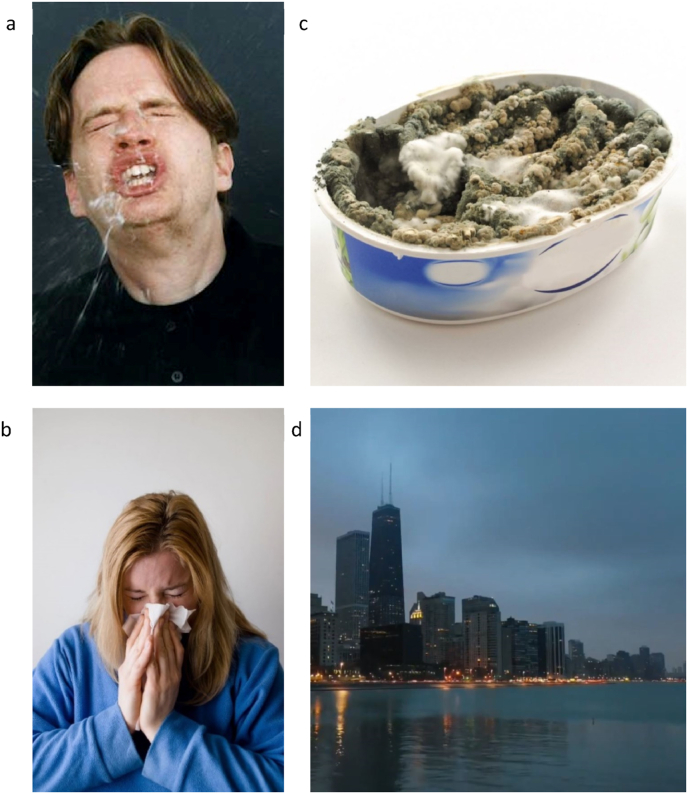
Examples from the four stimulus sets: a) Aerosol-Primer (© Ulf Lundin); b) Concealed Contagion-Primer (Mojep (pixaby.com)); c) Core Disgust-Primer (Haberkamp et al., 2017); d) Control-Primer (Ricardo Esquivel (pexels.com)).

**Fig. 2:**
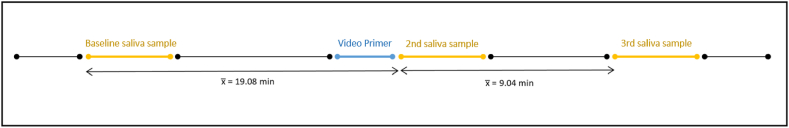
Timing of the test session: Questionnaires (black), saliva samples (yellow) and video primer (blue) in relation to each other during the experiment. Average time between the starting points of the saliva samples is indicated below the chart. (For interpretation of the references to colour in this figure legend, the reader is referred to the Web version of this article.)

**Fig. 3:**
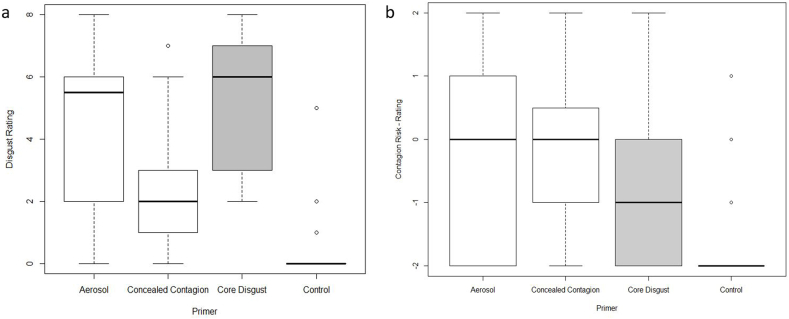
Post-video evaluation of Disease (white), Disgust (grey) and Control Video Primers with regard to a) disgust rating (“*How strong was your feeling of disgust, antipathy and revulsion while watching the video?*”; 0 = *”not at all”*, 8 = *”completely”*) and b) contagion risk rating (“*If this was a real situation I would have become sick.*” 0 = *”completely incorrect”*; 4 = *”completely correct”*).

**Fig. 4:**
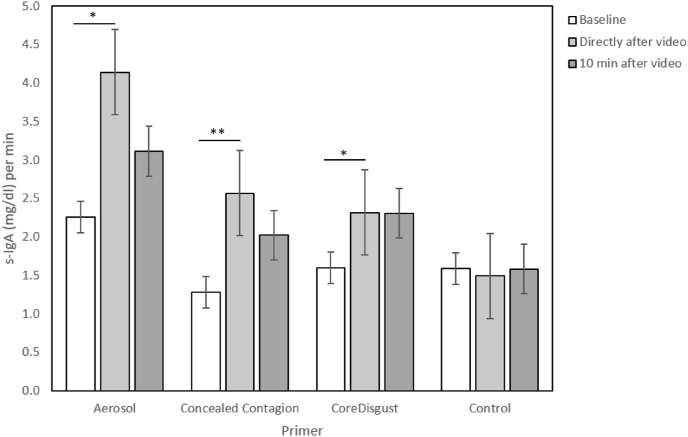
Mean and standard error of the S-IgA concentration by primer at Baseline, Sample 2 and Sample 3. Significant changes are marked with asterisks (*p < .05; **p < .01).

### 4.1 Influence of state disgust and perceived contagion risk on S-IgA

**Fig. 5:**
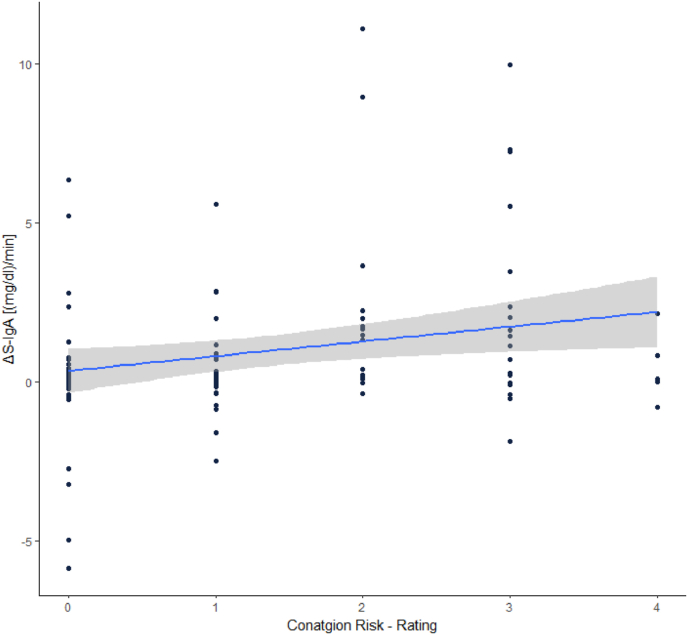
Correlation between ΔS-IgA and contagion risk rating (“*If this was a real situation I would have become sick.*” 0 = *”completely incorrect”*; 4 = *”completely correct”*).

We correlated the rating of how disgust evoking presented stimuli were from (1.) the Evaluation of Stimuli, and (2.) the mDES (“*How strong was your feeling of disgust, antipathy and revulsion while watching the video?*”) with the ΔS-IgA. This was done to investigate if state disgust, evoked by the presented videos, was related to the increase in S-IgA concentration. We found no significant correlations between the two state disgust measures and the ΔS-IgA (Video evaluation: r_s_ = 0.040, p = .683; mDES question: r_s_ = 0.045, p = .644).Furthermore, we correlated the above-mentioned rating of the perceived contagion risk with the increase of S-IgA. Here, we found a significant correlation (r _s_ = 0.230, p = .018), which is displayed in Fig. 5.

**Fig. 6:**
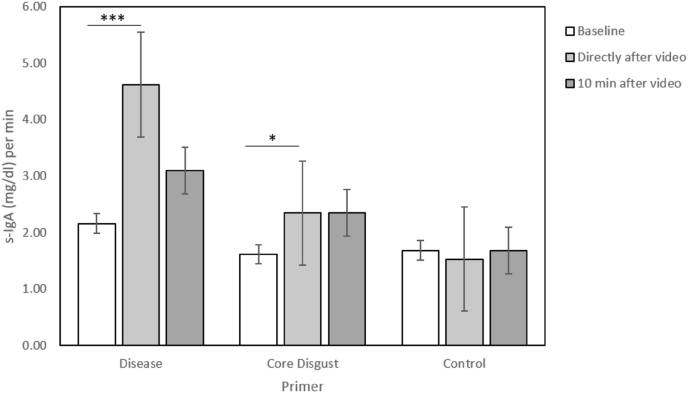
Subsample of the participants who perceived the disease videos as potentially contagious, in comparison to the core disgust and control primers. Mean and standard error of S-IgA concentration at Baseline, Sample 2 and Sample 3. Significant changes are marked with asterisks (*p < .05; ***p < .001).

In fact, only a subgroup of the participants, who watched the two disease-related primers perceived the video content as a realistic contagion risk, hinting that the video material might not have been realistic enough for each observer. This finding led us to conduct a secondary analysis, in which we excluded participants from the analysis, who did not rate the disease video primers as a potential contagion risk (n = 25). In addition, we also excluded participants, who in turn perceived the control primer as a contagion risk (n = 3). After excluding these 28 participants, we combined the two disease-related primers (Aerosol and Concealed Contagion) to one Disease Primer (n = 28), in order to keep the sample size comparable to that of the other two primer groups. The correlation between contagion risk and ΔS-IgA stayed significant after exclusion of these cases (r_s_ = .255, p = .023). Running the same GLM as before, we still found the significant main effect of Sample (F(2, 76) = 11.32, p = .001, η_p_^2^ = 0.130) and the interaction between Sample and Primer (F(1,76) = 6.64, p = .002, η_p_^2^ = 0.149, Fig. 6).

**Fig. 7:**
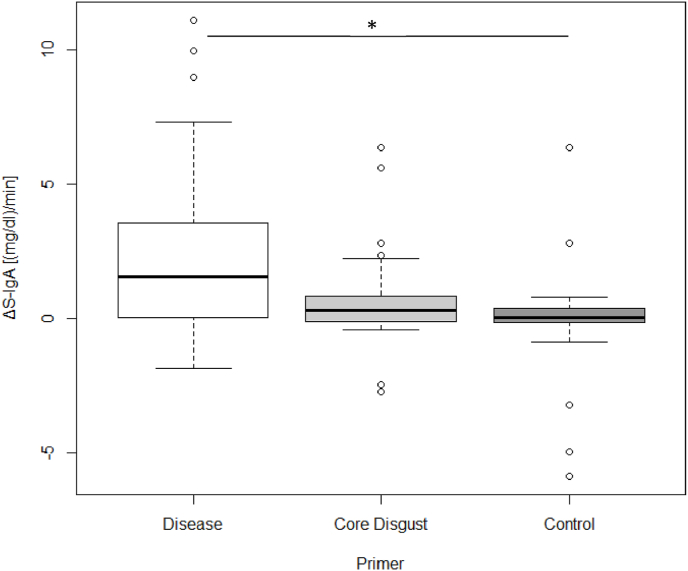
Subgroup analysis of participants who perceived the disease-related primers as potentially contagious. ΔS-IgA concentration represents the increase between Baseline and Sample 2 (Sample 2 – Baseline). The significant difference is marked with an asterisk (*p < .05).

Using the same post-hoc analysis we found a significant increase in the disease- and disgust-related primer (Disease: z = 3.48, p < .001; CD: z = 2.21, p = .027; C: z = 0.417, p = .677, Fig. 6, Supplementary Table 6). (ΔS-IgA) we found that the increase in S-IgA was significantly higher after the Disease Primer than after the Control Primer (U = 190.00, p = .004, p_a_ = .012, η = 0.153, Fig. 7). However, the S-IgA increase to the potentially contagious video content still did not significantly differ from the increase evoked by the Core Disgust Primer (U = 266.50, p = .091, p_a_ = .273, η = 0.053), which yet did not differ from the Control Primer (U = 240.50, p = .111, p_a_ = .333, η = 0.050).

## 5 Influence of trait disgust and perceived vulnerability to disease on S-IgA

**Fig. 8:**
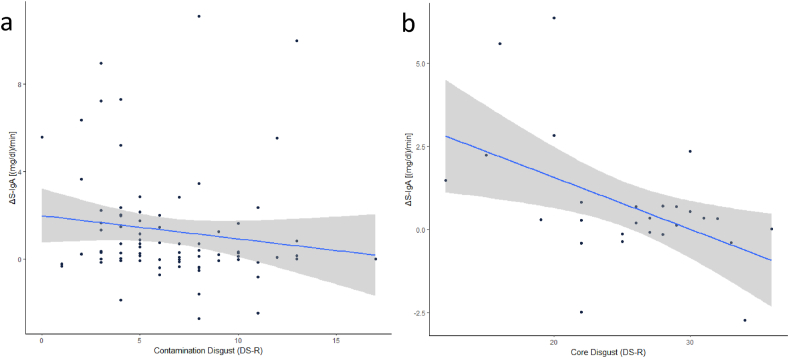
Correlation between a) ΔS-IgA and Contamination Disgust (DS-R), excluding the control primer and b). ΔS-IgA and Core Disgust (DS-R) in participants that watched the core disgust stimuli.

Investigating the relationship between the S-IgA increase after the disease- or disgust-related primers and (1.) trait disgust, and (2.) VtD, we correlated the ΔS-IgA to the DS-R and pVtD scales and subscales. Since the control group showed no significant increase in S-IgA, we excluded these participants from the analyses. While we found no significant correlation between the ΔS-IgA and the total DS-R (r _s_ = −.187, p = .097) or the Core Disgust subscale (r_s_ = -.144, p = 203), we found a significant negative correlation with the Contamination Disgust subscale (r_s_ = -.239, p = .033, Fig. 8a).

As the core disgust subscale mainly targets the items that are represented in the core disgust video, we also correlated the core disgust subscale separately to each primer. We found a significant correlation between the ΔS-IgA and core disgust in participants that were presented with the core disgust video (r_s_ = -.338, p = .046, see Fig. 8b), while the other groups showed no significant correlation (A: r_s_ = 0.158, p = .441; CC: r_s_ = -.206, p = .302). Furthermore, we did not find a significant correlation between the increase of S-IgA and the pVtD sum scale (r_s_ = -.039, p = .728) or its subscales Germ Aversion (r_s_ = 0.062, p = .583) and Perceived Infectability (r_s_ = -.104, p = .356).

## 6 Discussion

The goal of our study was to get a better insight into the interaction of the behavioral and the physiological immune system in disease- and disgust-related contexts. For this purpose, we created four sets of realistic video stimuli, which differed in their disease-association/contagion risk and overall disgust potential. We further measured the increase of S-IgA as evoked by the different videos and utilized various state and trait measures that revealed individual differences in disgust sensitivity and perception of disease threats. We found a significant increase of S-IgA secretion in our three disease- and disgust-related stimuli, which correlated positively with the perceived contagion risk and inversely with a trait measure of contamination disgust. On average, the S-IgA concentration increased by 83.15% after the Aerosol, by 100.63% after the Concealed Contagion and by 44.79% after the Core Disgust Primer. This was in so far unexpected since prior studies either reported only slight increases that occurred under specific circumstances (e.g., only in people with high trait disgust, Stevenson et al., 2015; or during a specific season, Brown et al., 2014) or even found a drop in S-IgA, particularly after disgust-evoking stimuli (Bosch et al., 2001; Stevenson et al., 2011, 2012). We can only speculate that the significant rise of S-IgA after all disease- and disgust-related videos was caused by the improved and supposedly more realistic stimulus material. On the one hand, we used short video clips showing real-life situations in combination with still pictures. On the other, our disease-related videos targeted respiratory pathogens and implied an increased risk of airborne contagion by the humans displayed. Previous studies used quite different disease-associated primers like x-rays or hospital hallways (Stevenson et al., 2015) or showed sick people who indicated no direct contagion risk (Brown et al., 2014). One may therefore assume that our videos more specifically activated defensive immune responses of the oral and nasal mucosae, which became evident in the significant rise of S-IgA in response to the two disease primers.

However, the extent of the actual increase (ΔS-IgA) induced by the two disease primers did not differ significantly from the control group, yet in the disease primer groups a significant rise from Baseline to Sample 2 could be documented which was not visible in the control primer (Fig. 4). Moreover, there was also no significant difference in the ΔS-IgA between the disease- and disgust-related primers. This lack of any significant differences in the primary analysis of the ΔS-IgA between primers could partially be caused by the data variance. Especially, the baseline sample showed a high variation between the four conditions, with the aerosol-group having the highest (x̅ = 2.26 [(mg/dl)/min]; σ = 2.23) and the concealed contagion group with the lowest standard deviation (x̅ = 1.28[(mg/dl)/min], σ = 1.38; also see Fig. 3). S-IgA is a very sensitive state measure and can be affected by many factors like psychological stress (Deinzer et al., 2000), physical stress (e.g.: sport, Keaney et al., 2018) and the consumption of certain foods and/or drinks (Kono et al., 2018). As our study was conducted during the COVID-19 pandemic we had to use an online test format, in which we were not able to control such factors up to the usual lab standard. We intentionally decided to use a between-subjects design for these ‘at-home tests’, since in a within-subject design with repeated tests over several days either the requirement of sample storage in participants' freezers over multiple days or the necessity to send the collected samples in multiple packages could have severely compromised sample quality. We are convinced that testing participants in a controlled laboratory environment with standardized relaxation periods for acute stress reduction and there also repeatedly with the different videos, i.e., in a within-subject design, would have significantly improved our data. The collection of stress related factors such as cortisol (Pawlow and Jones, 2005) as well as individual health parameters such as the level of B cells (Salvi and Holgate, 1999) might give an even better understanding of the variation in S-IgA concentrations and should be considered in a future between-subject design. Lastly, contributing to the heightened variance in the data, the present study design could have been slightly confounded. The participants filled in both the DS-R and the pVtD between the Baseline and the second (post-video) saliva sample and before watching the video primer. The statements of these questionnaires confront the participants with disgust-as well as disease-related situations, which might have had a promoting effect on S-IgA, even before the stimulation by the videos occurred. This seems plausible, since emotionally charged and disgusting statements alone have been found to already trigger disgust-related activation in the brain (Moll et al., 2005).

Furthermore, we found that the perceived contagion risk associated with the videos correlated positively to the increase of S-IgA, implying that perceived contagion risk is an important factor in defensive immune responses. This assumption led us to perform a secondary analysis, from which we excluded participants that did not see the disease-related primers as a realistic contagion risk. Here we found a significant difference in ΔS-IgA between the disease-related and control primers, while the core disgust primer still did not significantly differ from neither the control nor the disease-related primer (Fig. 7). Lastly, it is important to mention that the data were collected during the Covid-19 pandemic. Although, the online tests were conducted from May to October 2021 during a period of relatively low case numbers, this pandemic of a potentially deadly respiratory disease, has altered the perception of sneezing and coughing people (Bouayed, 2022). Thus it might have had an influence on the extent of the S-IgA secretion, which needs to be ascertained by future post-pandemic replications. When looking into the interaction between BIS and PIS we could not find support for a co-occurrence of the two (high BIS activation = high physiological immune response), like Stevenson and colleagues found (2015). Contrary, our data show that the DS-R subscale contamination disgust correlated inversely with ΔS-IgA (Fig. 8a), and this was also the case for the core disgust DS-R subscale in participants that viewed the core disgust video (Fig. 8b). This would rather support the theory that the BIS and PIS compensate for each other (as also suggested by Fleischman and Fessler, 2011; Gassen et al., 2018). We therefore assume that people with a weaker PIS response, as reflected by a reduced ΔS-IgA after stimulation, might compensate this whenever confronted with a sick person by perceiving the situation as generally more disgusting, which also triggers increased avoidance, hence lowering contagion risk (Campbell et al., 2020; Dorfan and Woody, 2011). It is important to note that the findings of Stevenson et al. (2015) were based on a different disgust inventory than the one we used. They found a positive correlation between the change in S-IgA (after the presentation of their disgust-image set) to the pathogen subscale from the three dimensional disgust scale (Tybur et al., 2009, TDDS). They found no correlation to neither the DS-R contamination nor the core disgust subscale in their data.Nevertheless, these scales are close enough to compare the results. The pathogen subscale is defined as measure for the avoidance of infectious microorganisms (Tybur et al., 2009), the core disgust subscale measures sensitivity to offensiveness and threat of diseases and the contamination disgust subscale represents perceived threat of disease transmission (Olatunji et al., 2007). When comparing the items of the three questionnaires, the pathogen subscale (body fluids, rotten foods and animals) seems to combine the DS-R subscales of core disgust (disgusting food, animals, low hygiene) and contamination disgust (infectious body fluids, direct contact with pathogens). We speculate that the finding of their correlation (Stevenson et al., 2015), did not even represent the actual increase of S-IgA, but a relative value to a negative image set (anger-evoking: guns, domestic violence, personal distress), cannot be considered as reliable, since it may be severely statistically underpowered. Nevertheless, the absence of a correlation between ΔS-IgA and pVtD and its subscales in our study is coherent with the findings of Stevenson et al. (2015).

Furthermore, we found no significant correlation between our measures of state disgust (i.e., disgust questions from the Video evaluation and the mDES) and the S-IgA increase. This is coherent with findings of previous studies measuring S-IgA (Stevenson et al., 2015), IL-6 (Schaller et al., 2010), TNF-a/albumin and body temperature (Stevenson et al., 2012). Self-reported state disgust might not be the best way to measure the BIS in direct confrontations with disgust- and disease-related stimuli, as self-report of current state is very subjective and explicit, which makes it vulnerable to confounds like demand effects. Implicit methods such as behavioral computer tasks (e.g., to measure disease cue avoidance) might be a more suitable measure since they rather pick up implicit differences in the behavioral immune responses.

## 7 Limitations and future directions

Our data show activation of the PIS (higher concentration of S-IgA in saliva) following visual exposure to disease- and disgust-related stimuli. While one may speculate that the increase in S-IgA reflects a proactive immune response, that may prepare the organism for the upcoming pathogens associated with the sneezes and coughs, our study does not provide direct evidence for a heightened immunity in individuals who responded with increased S-IgA to the respective videos. However, such a proactive defense mechanism seems likely, since S-IgA in saliva plays an important role in immune exclusion and intracellular neutralization (Corthésy, 2013; Strugnell and Wijburg, 2010). Adding to that, heightened S-IgA is also discussed as a central biomarker of a reduced vulnerability to upper respiratory infections (Turner et al., 2021). Future studies have to further assess whether this increase in S-IgA represents the actual initiation of a preparatory immune response and thus reflects heightened immunity against the most common respiratory viruses even before the mucosae have come in contact with a pathogen. Nevertheless, we see our video priming experiment as a suitable method to further investigate how this interaction between the PIS and BIS is mediated by the Neuro-Immune-Axis in future neuroimaging studies.

## 8 Conclusion

Our findings are indicative of an enhanced activation of salivary S-IgA secretion through visual perception of potentially contagious objects and people. We also found that people with a high contamination disgust secreted less S-IgA in such situations, which might be a hint for a compensating relationship between BIS and PIS. To get a further insight into the association of the BIS and PIS more research is needed. For future studies we suggest a better baseline control to reduce variance in data. This might be done by testing the participants in the controlled environment of a laboratory, by letting them relax for some minutes before the baseline sample and putting stricter restrictions on participants before testing (no spicy food on the day, no caffeine, etc.). We also suggest the implementation of additional implicit behavioral measures (e.g., implicit avoidance tasks) to get a better understanding of the behavioral response to the presented disease stimuli, since this aspect of the BIS may be insufficiently be represented by self-report questionnaires alone.

## Declaration of interest

None.

## Role of funding

This research was funded by the regular research budget of the Neuroendocrinology and Human Biology Unit, at the 10.13039/501100005711Universität Hamburg.

## Declaration of interests

The authors declare that they have no known competing financial interests or personal relationships that could have appeared to influence the work reported in this paper.
