# Supplementary material for: Disease-related disgust promotes antibody release in human saliva
Source: Brain Behav Immun Health. 2022 Jul 14;24:100489. doi: 10.1016/j.bbih.2022.100489 (PMC9293731; doi:10.1016/j.bbih.2022.100489)
Supplement: Multimedia component 1 [file mmc1.docx]

Supplementary
Supplementary Table 1: Sequence (Seq.), Kind, Length, Description and mean from Evaluation of Stimuli (EoS) of each stimulus within the Aerosol Disease Primer.

| Seq. | Kind | Length (s) | Description | EoS |
| --- | --- | --- | --- | --- |
| 1 | Photo | 4 | Women, facing camera, sneezing, aerosols visible | 4.10 |
| 2 | Video | 27 | Men, side profile, slow motion, sneezing, aerosols visible | 4.04 |
| 3 | Video | 9 | Women, facing camera, sneezing, teary eyes | 2.36 |
| 4 | Photo | 4 | Child, male, side profile, sneezing, aerosols visible | 3.73 |
| 5 | Video | 8 | Men, facing camera, sneezing | 2.78 |
| 6 | Video | 3 | Men, facing camera, sneezing, drool and aerosols visible | 2.00 |
| 7 | Video | 4 | Men, facing camera, sneezing | 2.12 |
| 8 | Photo | 4 | Men, side profile, sneezing, aerosol visible | 4.03 |
| 9 | Video | 13 | Women, side profile, slow motion, sneezing, aerosol visible | 2.41 |
| 10 | Photo | 4 | Men, facing camera, sneezing, a lot of visible aerosol | 4.81 |
| total |  | **80** | **Video was shown twice without any breaks** | **3.24** |

Supplementary Table 2: Sequence (Seq.), Kind, Length, Description and mean from Evaluation of Stimuli (EoS) of each stimulus within the Concealed Contagion Disease Primer.

| Sequence | Kind | Length (s) | Description | EoS |
| --- | --- | --- | --- | --- |
| 1 | Photo | 4 | Men, facing camera, sneezing into right elbow | 1.82 |
| 2 | Video | 27 | Men, walking towards camera, sneezing into hand | 1.77 |
| 3 | Video | 9 | Women, side profile, sneezing into tissue, blowing nose | 2.17 |
| 4 | Photo | 4 | Women, facing camera, blowing nose into tissue | 1.91 |
| 5 | Video | 15 | Women, side profile, in bed, coughing/ blowing nose | 1.57 |
| 6 | Photo | 4 | Women, side profile, coughing into fist | 1.86 |
| 7 | Video | 13 | Men, facing camera, on couch, sneezing into tissue | 2.23 |
| 8 | Photo | 4 | Women, facing camera, blowing nose into tissue | 2.47 |
| total |  | **80** | **Video was shown twice without any breaks** | **1.98** |

Supplementary Table 3: Sequence (Seq.), Kind, Length, Description and mean from Evaluation of Stimuli (EoS) of each stimulus within the Core Disgust Primer.

| Sequence | Kind | Length (s) | Description | EoS |
| --- | --- | --- | --- | --- |
| 1 | Photo | 4 | Mold on cream cheese container | 4.16 |
| 2 | Video | 27 | Several rats, dirty floor, dirty feet | 3.76 |
| 3 | Video | 9 | Several hornet larva | 2.97 |
| 4 | Photo | 4 | Cockroach | 2.45 |
| 5 | Video | 9 | Dog vomiting grass | 2.06 |
| 6 | Video | 6 | Mold on bread slices | 3.69 |
| 7 | Photo | 4 | Very dirty and overly messy room | 3.5 |
| 8 | Video | 13 | Strawberries molding, slow motion | 3.78 |
| 9 | Photo | 4 | Dead bat, with larva spilling out of open gut | 4.93 |
| total |  | **80** | **Video was shown twice without any breaks** | **3.48** |

Supplementary Table 4: Sequence (Seq.), Kind, Length, Description and mean from Evaluation of Stimuli (EoS) of each stimulus within the Control Primer.

| Sequence | Kind | Length (s) | Description | EoS |
| --- | --- | --- | --- | --- |
| 1 | Photo | 4 | Bottom of plane flying over skyscrapers | 0.68 |
| 2 | Video | 27 | Drone flight over Chicago skyline | 0.53 |
| 3 | Video | 9 | Drone flight around skyscraper | 0.64 |
| 4 | Photo | 4 | Yellow entrance door to apartment building | 0.64 |
| 5 | Video | 9 | Pan over park with lake | 0.37 |
| 6 | Video | 6 | Birdseye view on fountain in the middle of a roundabout | 0.80 |
| 7 | Photo | 4 | Bike at bottom of a long bridge (over a river) | 0.60 |
| 8 | Video | 13 | Drone flight over skyscraper | 0.94 |
| 9 | Photo | 4 | Subway in train station | 1.04 |
| total |  | **80** | **Video was shown twice without any breaks** | **0.69** |

Supplementary Table 5: All questions of the Relative-Feelings Questionnaire with phrasing of question in German.

|  | Question |
| --- | --- |
| 1 | Relative to before I feel more uneasy or apprehensive.  (Ich fühle mich relative zu vorher unwohler oder beklommener.) |
| 2 | Relative to before I feel more inspired or creative.  (Ich fühle mich relative zu vorher inspirierter und kreativer.) |
| 3 | Relative to before I feel more stressed or burdened.  (Ich fühle mich relative zu vorher gestresster oder belasteter.) |
| 4 | Relative to before I feel more amused or exhilarated.  (Ich fühle mich relative zu vorher amüsierter oder beschwingter.) |
| 5 | Relative to before I feel weaker or sicker.  (Ich fühle mich relative zu vorher schwächer oder kränklicher.) |
| 6 | Relative to before I feel more optimistic or energetic.  (Ich fühle mich relative zu vorher optimistischer und tatkräftiger.) |

Supplementary Table 6: Subgroup analysis of participants who perceived the disease-related primers as potentially contagious. Descriptive statistics of the comparison of S-IgA [(mg/dl)/min] between Baseline and Sample 2 for the different primers.

| **Stimuli** | **n** | **x̅_Baseline_** | **x̅_Sample2_** | **σ_Basline_** | **σ_Sample2_** | **z** | **p** |
| --- | --- | --- | --- | --- | --- | --- | --- |
| Disease | 28 | 2.16 | 4.62 | 2.16 | 4.88 | 3.48 | >.001 *** |
| Core Disgust | 27 | 1.60 | 2.31 | 1.72 | 2.79 | 2.21 | .027 * |
| Kontrolle | 24 | 1.69 | 1.53 | 2.49 | 1.61 | .47 | .677 - |

Supplementary Table 7: Descriptive data on Sex differences in trait Disgust (DS-R and subscales), p-Values are based on Mann-Whitney-U test between the sexes.

|  | Sum Disgust | | Contamination Disgust | | Core Disgust | |
| --- | --- | --- | --- | --- | --- | --- |
| sex | f | m | f | m | f | m |
| N | 68 | 48 | 68 | 48 | 68 | 48 |
| Mean | 34.71 | 29.77 | 6.68 | 6.40 | 28.03 | 23.38 |
| p-value | .004 | | .503 | | >.001 | |

Supplementary Table 8: Descriptive data on Sex differences in trait vulnerability to disease (VtD), p-Values are based on Mann-Whitney-U test between the sexes.

|  | Sum VtD | | Germ Aversion | | Preceived Infectability | |
| --- | --- | --- | --- | --- | --- | --- |
| sex | f | m | f | m | f | m |
| N | 68 | 48 | 68 | 48 | 68 | 48 |
| Mean | 49.97 | 49.63 | 27.79 | 26.81 | 22.18 | 22.81 |
| p-value | .877 | | .497 | | .606 | |


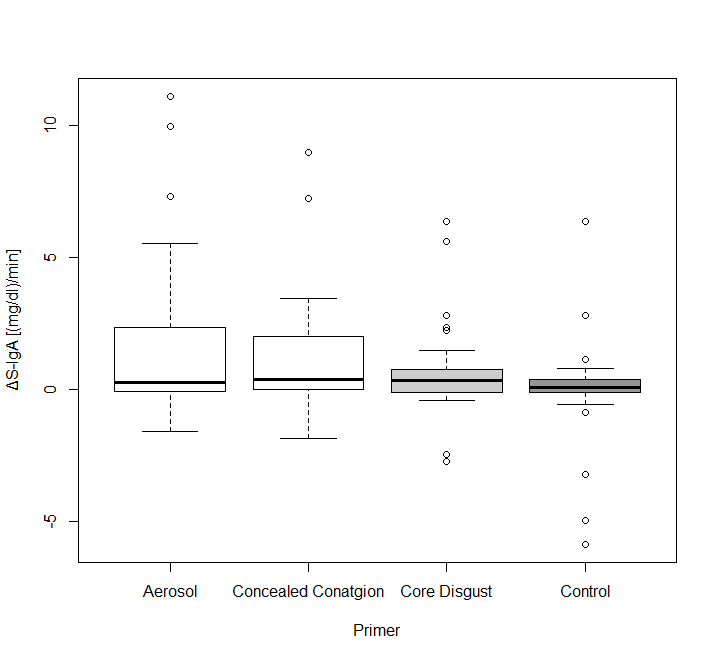


Supplementary Figure 1: ΔS-IgA concentration between Baseline and Sample 2, according to primer.


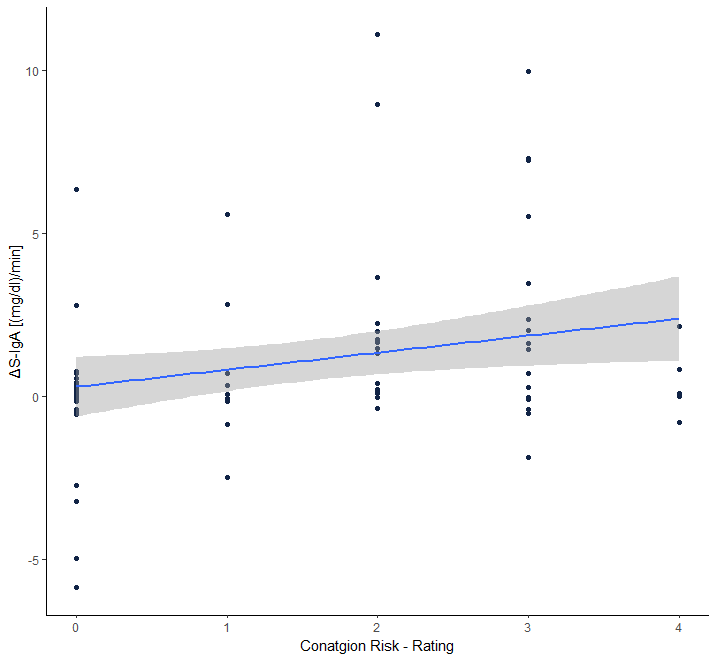


Supplementary Figure 2: Subgroup analysis of participants who perceived the disease-related primers as potentially contagious. Correlation between ΔS-IgA and perceived Contagion Risk (rating after the video).
